# Methamphetamine Compromises the Adaptive B Cell-Mediated Immunity to Antigenic Challenge in C57BL/6 Mice

**DOI:** 10.3389/ftox.2021.629451

**Published:** 2021-03-15

**Authors:** Anum N. Mitha, Daniela Chow, Valerie Vaval, Paulina Guerrero, Dormarie E. Rivera-Rodriguez, Luis R. Martinez

**Affiliations:** 1 Department of Biomedical Sciences, Long Island University, Brookville, NY, United States; 2 Department of Biomedical Sciences, NYIT College of Osteopathic Medicine, New York Institute of Technology, Old Westbury, NY, United States,; 3 Department of Biological Sciences, The Border Biomedical Research Center, The University of Texas at El Paso, El Paso, TX, United States; 4 Department of Biology, University of Puerto Rico-Ponce, Ponce, PR, United States; 5 Department of Oral Biology, University of Florida College of Dentistry, Gainesville, FL, United States

**Keywords:** ovalbumin, methamphetamine, lipopolysaccharide, B cells, antibodies

## Abstract

Methamphetamine (METH) is a substance of abuse that causes dysregulation of the innate and adaptive immunity in users. B cells are involved in the humoral component of the adaptive immunity by producing and secreting antibodies (Abs). METH modifies Ab production, although limited information on the impact of this psychostimulant on antigen (Ag)-specific humoral immune responses is available. Since T cell-dependent and T cell-independent Ags are involved in the activation of B lymphocytes, we explored the role of METH on humoral immunity to ovalbumin (OVA; T cell-dependent) and bacterial lipopolysaccharide (LPS; T cell-independent) in C57BL/6 mice. We demonstrated that METH extends the infiltration of B cells into pulmonary and splenic tissues 7 days post-Ag challenge. METH impairs Ab responses in the blood of animals challenged with OVA and LPS. Furthermore, METH diminishes the expression and distribution of IgM on B cell surface, suggesting a possible detrimental impact on users’ humoral immunity to infection or autoimmunity.

## INTRODUCTION

Methamphetamine (METH) is an addictive substance of abuse that stimulates the central nervous system considerably. The public health and economic burden posed by the use of METH is significant globally (The RAND Corporation, 2009), particularly in the United States (U.S.). It is estimated that ~1.6 million people (0.6% of the U.S. population) use METH each year, and ~774,000 (0.3% of the U.S. population) use it every month (NIDA, 2019). In fact, METH consumption in the U.S. has also augmented 7.5 times in the last decade (NIDA, 2019). METH is currently responsible for ~15% of all overdose-related deaths in the U.S. and half of the deaths involve opioids (Hedegaard et al., 2018). METH causes a quick increase in dopamine release in areas of the brain associated with reward functions, promotes drug consumption repetitiveness, and increases dependence (Battaglia et al., 2002; Calabresi et al., 2007; Homer et al., 2008; Volkow and Morales, 2015).

METH affects the behavior of users and make them prone to get involved in health-related risky activities including having sex without protection and contaminated needle exchange with other users, resulting in infectious disease acquisition (Ellis et al., 2003). METH facilitates the transmission of HIV (Ellis et al., 2003), hepatitis virus (Gonzales et al., 2006), *Mycobacterium tuberculosis* (Mankatittham et al., 2009), herpes virus (Valencia et al., 2012), and many other pathogens (Galindo et al., 2012). METH impairs the host innate and adaptive immunity (Saito et al., 2008; Martinez et al., 2009; Peerzada et al., 2013). METH causes defects in phagocyte migration and antimicrobial efficacy (Mihu et al., 2015). Likewise, METH reduces T cell populations and proliferation in mice (Freire-Garabal et al., 1991; Martinez et al., 2009; Peerzada et al., 2013). METH causes oxidative damage to the mitochondria and dysregulate the function of human T cells in culture (Potula et al., 2010). METH induces a mixed protective Th1 and a detrimental Th2 immune response facilitating infection and disease (Martinez et al., 2009). Moreover, METH exposure results in apoptosis in T lymphocytes (Potula et al., 2010) and macrophages (Aslanyan et al., 2019).

The abundant METH accumulation in most body organs in humans possibly contributes to the medical complications related to its dependance. In humans, the highest METH uptake occurs in the lungs (Volkow et al., 2010), increasing users’ susceptibility for respiratory infections (Mankatittham et al., 2009; Martinez et al., 2009). METH accumulates (Shiue et al., 1993; Fowler et al., 2007) and causes apoptosis (Iwasa et al., 1996) in the spleen, a critical organ for harboring B cells, which are responsible for the humoral immunity. The principal functions of B cells are antigen (Ag) presentation, differentiation into plasma cells, Ab production and release, and memory development. Although METH modifies Ab production during infection in rodents (In et al., 2005; Wey et al., 2008; Martinez et al., 2009), there is insufficient information on the impact of METH on humoral immunity *in vivo* upon antigenic (Ag) challenge.

Given that activation of B cells occurs by T cell-dependent and T cell-independent mechanisms, we investigated the effect of METH on B cell-mediated immune responses to ovalbumin (OVA) and bacterial lipopolysaccharide (LPS). OVA is a T cell-dependent Ag and presentation of OVA-class II MHC complex on a B cell allow these lymphocytes to become Ag-presenting cells to T cells. T cell receptors on the surface of T helper cells recognize and bind to the OVA-complexed class II MHC molecule on the B lymphocyte surface leading to the activation of T cells. A second activation signal occurs after the activated T cell through various proteins interacts with the B cell. After becoming activated, B cells replicate and form germinal centers where differentiation into memory or Ab-producing lymphocytes takes place. Upon plasma cell differentiation occurs, cytokine production stimulates plasma cell Ab class switching and production control. Alternatively, LPS is a T cell-independent Ag, which activates murine B cells without participation by T or other cells. T cell-independent Ags do not stimulate immunological memory.

In this study, we hypothesized that METH alters B cell infiltration and Ab responses in C57BL/6 mice after Ag challenge. Our objective is to understand the impact of METH on humoral immunity *in vivo*, which may be important in combating infectious diseases and the development of autoimmunity in users. Stimulation or inhibition of adaptive humoral immune responses represents a potential therapeutic strategy to combat METH-related infections or minimize autoimmunity in individuals at risk.

## MATERIALS AND METHODS

### Mouse Model of Acute METH Administration

METH addicts begin consuming small quantities of this substance of abuse occasionally before gradually increasing the dose (Simon et al., 2002). To mimic this behavior, increasing doses (2.5, 5, and 10 mg/kg/day on weeks 1, 2, and 3, respectively) of METH (Sigma) were administered daily intraperitoneally (i.p.) to female C57BL/6 mice (age, 6–8 weeks; Charles Rivers) for 21 days. Phosphate-buffered saline (PBS; untreated)-treated rodents were used as controls. Although biological sex differences in studies involving substances of abuse are important, we only used female mice in this study because splenic lymphocytes isolated from male and female rodents injected with a single dose of 5 mg/kg of METH showed no difference in their proliferation after exposure to 40 μg/mL of LPS (Saito et al., 2008). After PBS or METH treatments, animals were injected with 100 mg/kg ketamine (Keta-set®; Henry Schein) and 10 mg/kg xylazine (Anased®; Henry Schein) of the anesthetic mixture and intranasally (i.n.) challenged with only one dose of 50 μg/mL of either OVA (main protein found in egg white; Sigma) or LPS (Sigma) in a 50 μL suspension. OVA was selected as a T-dependent Ag because is frequently used as a model protein to study Ag-specific immune responses in mice. To rule out possible cross-contamination, the OVA solution was evaluated for the presence of bacterial LPS before each injection using an endotoxin detection kit (InvivoGen). On days 3 and 7 post-Ag challenge, each mouse was anesthetized, bled, and euthanized. Then, the organs were removed to analyze the role of METH on B cell responses. The right and left lungs of each mouse were weighed and collected for flow cytometry and histology, respectively. Each spleen was divided into two similar size halves and each half of tissue was weighed and utilized for flow cytometry and histology. Naïve mice were also used as controls. All the untreated and METH-treated animals survived the injection regime and Ag challenge. Mice were kept in a controlled environment with temperature between 22 and 24.5°C with a 12:12-h light/dark cycle and *ad libitum* access to food and water. All animal studies were conducted according to the experimental practices and standards approved by the Institutional Animal Care and Use Committee at the NYIT College of Osteopathic Medicine (Protocol #: 2016-LRM-01).

### Rationale for METH Doses Used in These Studies

A single 260-mg dose of METH increases at a level of 7.5 μM (Melega et al., 2007). Thus, after METH injection with 260 mg the levels of the drug in blood range between 7.5 and 28.8 μM. Multiple injections of METH results in higher drug levels because this psychostimulant has a half-life of ~12 h (Cho et al., 2001; Harris et al., 2003). Users with four consecutive injections with 260 mg in the same day show 17 μM in blood that can reach 20 μM 2 days after binging (Melega et al., 2007). Thus, binge doses between 260 and 1,000 mg result in 17–80 μM of METH in blood while drug levels can reach μm levels in the hundreds in body organs such as the brain and the spleen (Cho et al., 2001; Harris et al., 2003). Hence, we used 25 μM METH to carry out all the experiments.

### Flow Cytometry Was Used to Determine B Cell Infiltration in Murine Tissue

Murine primary cells were obtained from 0.1 g of excised and homogenized lung and spleen tissue in 1 mL PBS from 5 mice treated with PBS, METH, OVA, METH + OVA, LPS, and METH + LPS as described above; the cells were washed, incubated with murine Fc seroblock (clone FCR4G8, dilution 1:10; BioRad) for 10 min to prevent non-specific binding of test Abs, and then stained with either CD45R (B cells; clone RA3–6B2; dilution: 1:1,000; Santa Cruz Biotechnology; SCB)-fluorescein isothiocyanate (FITC)-labeled Ab or its corresponding isotype controls for 1 h at 37°C. Each sample was handled on a BD Accuri C6 flow cytometer and analyzed utilizing the FCS Express software version 4, and the number of CD45R^+^ cells per 0.1 g of tissue was reported per condition.

### Histology

Upon METH injection and Ag sensitization, each mouse was sacrificed and lung/spleen tissues were removed and fixed in 4% paraformaldehyde (Sigma) for 24 h. Tissues were managed, embedded in paraffin, and 4 μm sagittal sections were fixed to glass slides. The tissues were then stained for B cells using a CD45R-specific Ab (dilution: 1:1,000; SCB) conjugated to horseradish peroxidase. Slides were observed using an Olympus BX41 inverted microscope and images were taken with an Olympus DP70 camera using Olympus DP Controller software version 3. Each image was blindly analyzed by two independent investigators. Ten microscopic fields per image were counted and the average was calculated per image (*n* = 10 images; 10 fields per image; 2 images per mouse; 5 mice per condition).

### Serum Ab Isotype Concentrations

Mice were anesthetized and 100 μL of retro-orbital blood collected. The blood was centrifuged at 10,000 × g for 10 min at 4°C to separate the serum from the blood cellular components. Then, the upper clear layer containing the serum was collected and stored at −20°C until analysis. Ab isotype concentration was measured by enzyme-linked immunosorbent assay (ELISA) and compared to isotype-matched standards. Ninety six-well microtiter plates (Corning) were coated with goat anti-mouse IgM, IgG_1_, IgG_2a_, IgG_2b_, or IgG_3_ (1 μg/mL; Southern Biotech) and blocked with 1% bovine serum albumin (BSA; Thermo Fisher; TF) in PBS. Then, serum was added and the ELISA was completed by adding, in successive steps, 1 μg of alkaline phosphatase-labeled goat-anti mouse IgM, IgG_1_, IgG_2a_, IgG_2b_, or IgG_3_/mL (Southern Biotech) and 50 μl of *p*-nitrophenyl phosphate (5 mg/mL; Sigma) in substrate buffer. Finally, the concentration of each isotype was determined with a microtiter reader (BioTek Epoch) at 405 nm. Between each step, the wells were washed with 0.05% Tween 20 in Tris-buffered saline. All incubations were done at 37°C for 1 h, at room temperature (RT) for 2 h, or at 4°C overnight.

### Human Burkitt Lymphoma B Cell Line (BJAB Cells)

BJAB cells were cultured in RPMI 1640 medium and supplemented with 15 % heat-inactivated fetal calf serum (FCS; Atlanta Biologicals), 100 U/mL penicillin (Gibco), 100 μg/ml streptomycin (Gibco), and 0.25 μg/ml amphotericin B (Gibco). Cells proliferating exponentially and confluency reaching ~60–75%, were counted, and cultured into 24-well-microtiter plates (Corning). The cells were incubated at 37°C and 5% CO_2_. For each experiment, 2 × 10^5^ BJAB cells were treated in absence or presence of 25 μM METH for 2 h, washed 3X with PBS, and incubated with 10 μg/mL of OVA or LPS for 24 h. Then, fluorescent microscopy, flow cytometry, and western blot analyses were performed.

### Fluorescent Microscopy

Fluorescent microscopy was performed and previously described in Vargas et al. (2020). Monolayers of BJAB cells were cultured on a glass-bottom petri dish (TF) and fixed with 4% formaldehyde (TF) for 20 min at RT. The cells were washed 3X with 5% Tween 20 (TF) in PBS and blocked with 1% BSA in PBS for 1 h at RT. After blocking, the cells were again washed 3X with 5% Tween 20 in PBS and incubated with an anti-IgM conjugated to Alexa Fluor® 488 (green; 1:100 dilution; SCB), in blocking solution in an orbital shaker (New Brunswick Galaxy 170S) at 150 rpm and 37°C for 1 h. The samples were washed 3X with blocking buffer and incubated with 4’, 6-diamidino-2-phenylindole (dapi; blue; TF) to stain nuclei for 1 h at 37°C. The slides were washed 3X with PBS, coverslips were affixed, and each sample was viewed to determine the IgM distribution on the surface of BJAB cells with a Zeiss LSM 700 Confocal Laser Scanning Microscope (Carl Zeiss) at a magnification of ×60. Images were collected using an AxioCam digital camera and analyzed using Zen Lite digital imaging software (Carl Zeiss).

### Statistical Analysis

All data were subjected to statistical analysis using Prism 8.0 (Graph Pad) as previously described (Vargas et al., 2020). *P*-values for individual comparisons were calculated by analysis of variance (ANOVA) and adjusted using the Tukey’s multiple comparison test. *P*-values of <0.05 were considered significant.

## RESULTS

### METH Maintains CD45R^+^ Cells Infiltration Into Lung and Splenic Tissue of C57BL/6 Mice 7 Days Post-challenge

**FIGURE 1 |:**
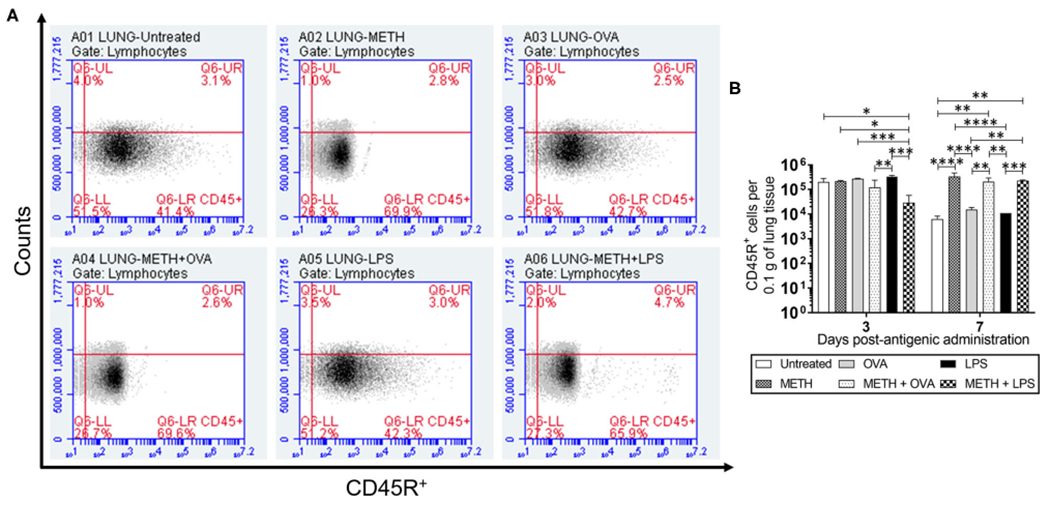
Methamphetamine (METH) extends CD45R^+^ cells infiltration into lung tissue of C57BL/6 mice 7 days after antigenic (Ag) challenge. Counts of B (CD45R^+^) cells per 0.1 g of **(A,B)** lung tissue of C57BL/6 mice (*n* = 5 mice per condition per day) 3- and 7-days post-METH and ovalbumin (OVA)/lipopolysaccharide (LPS) administration were analyzed by flow cytometry. Representative plots of pulmonary tissue for day 7 post-Ag challenge and bars indicate the average number of CD45R^+^ cells (*n* = 5 mice per condition per day) for untreated (PBS), METH, OVA, METH + OVA, LPS, and METH + LPS and error bars indicate standard deviation (STDEV). Asterisks (*) indicate *P*-value significance (*P < 0.05, **P < 0.01, ***P < 0.001, and ****P < 0.0001) calculated using analysis of variance (ANOVA) and adjusted by use of the Tukey’s *post-hoc* analysis. The experiments shown were performed twice, and similar results were obtained each time.

**FIGURE 2 |:**
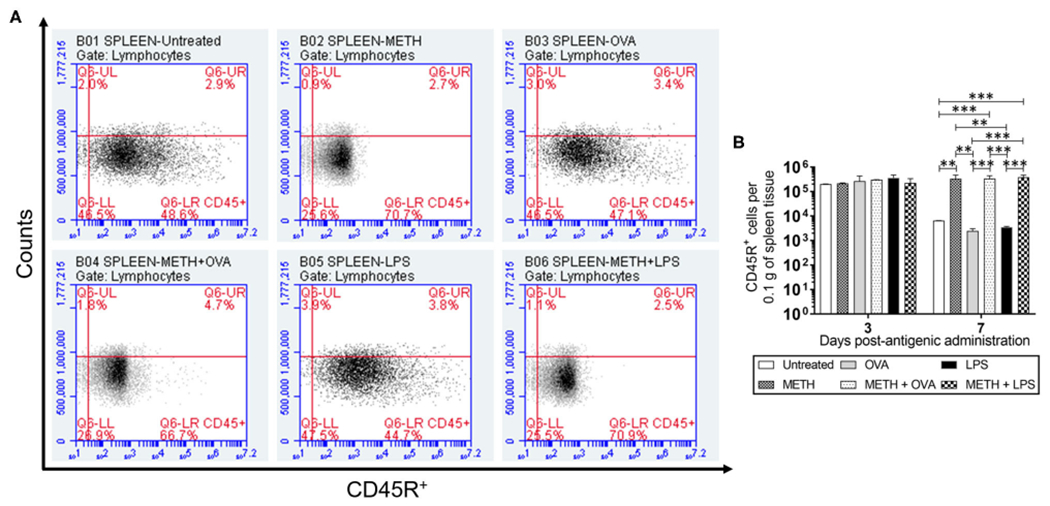
METH prolonged CD45R^+^ cells infiltration into splenic tissue of C57BL/6 mice 7 days after Ag challenge. Counts of B (CD45R^+^) cells per 0.1 g of **(A,B)** splenic tissue of C57BL/6 mice (*n* = 5 mice per condition per day) 3- and 7-days post-METH and OVA/LPS administration were analyzed by flow cytometry. Representative plots of splenic tissue for day 7 post-Ag challenge and bars indicate the average number of CD45R^+^ cells (*n* = 5 mice per condition per day) for untreated, METH, OVA, METH + OVA, LPS, and METH + LPS and error bars indicate STDEV Asterisks (*) indicate *P* value significance (**P < 0.01 and ***P < 0.001) calculated using ANOVA and adjusted by use of the Tukey’s *post-hoc* analysis. The experiments shown were performed twice, and similar results were obtained each time.

CD45R or B220, a biomarker for B cells in mice (CD19 in humans), is crucial in starting intrinsic B cell signaling thresholds by controlling both B cell receptor-dependent and independent signaling (Wang et al., 2012). Since B lymphocyte activation occurs either with or without T cells, we investigated the effect of METH on CD45R^+^ cell infiltration into the lungs (Figures 1A,B) and spleen (Figures 2A,B) of C57BL/6 mice challenged with OVA (T cell-dependent Ag) and LPS (T cell-independent Ag) using flow cytometry. On day 3 post-Ag administration, the lungs of mice sensitized with LPS had significantly higher CD45R^+^ cell infiltration than rodents treated with METH + OVA (*P* < 0.01) and METH + LPS (*P* < 0.001) (Figure 1B). Animals injected with METH + LPS displayed less CD45R^+^ cell recruitment in pulmonary tissue compared to the METH (*P* < 0.05) and OVA (*P* < 0.001) groups (Figure 1B). There were no differences in CD45R^+^ cell infiltration in splenic tissue 3 days post-Ag administration between the experimental groups (Figure 2B). On day 7 post-Ag administration, all the groups treated with METH evinced sustained and significantly higher CD45R^+^ cell infiltration into the lung (Figures 1A,B) and splenic (Figures 2A,B) tissue than those not treated with the drug (*P* < 0.01).

**FIGURE 3 |:**
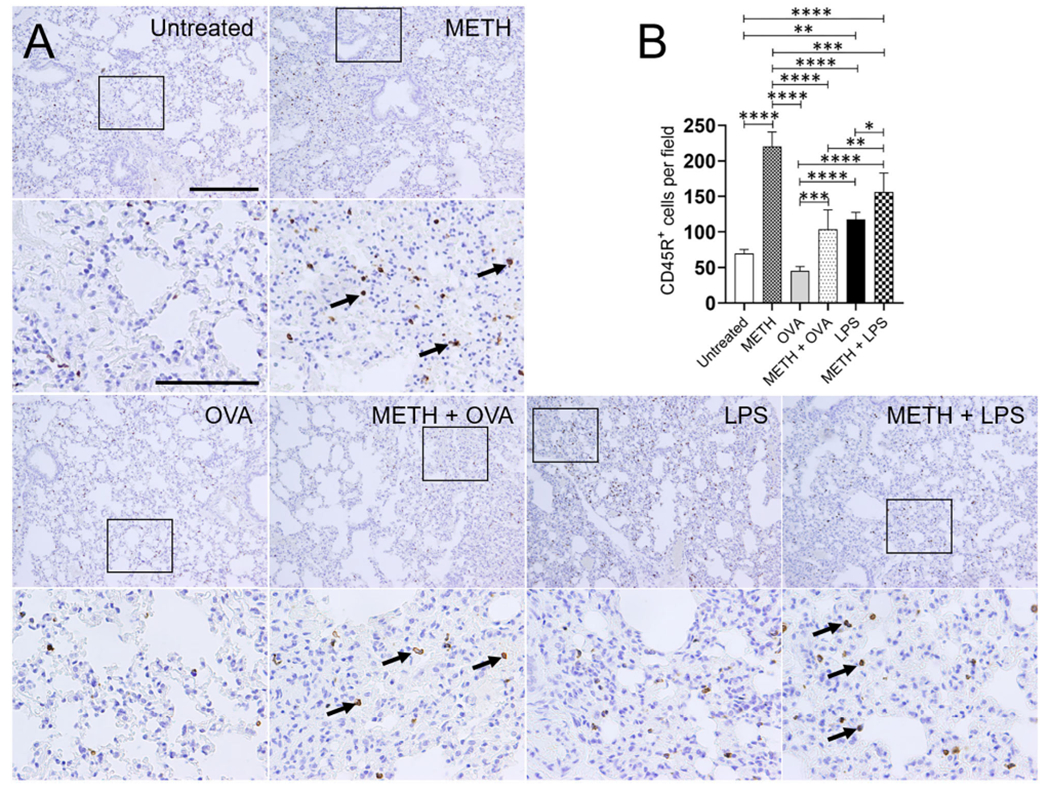
METH alters CD45R^+^ cells infiltration into lung tissue of C57BL/6 mice after sensitization with ovalbumin (OVA) or lipopolysaccharide (LPS). **(A)** Representative CD45R stained sections of lung tissue excised from untreated, METH, OVA, METH + OVA, LPS, and METH + LPS-treated animals after 7 days are shown (scale bar, 200 μm; 10 and 40x magnification). Black rectangular box delineates the area magnified. Brown staining (black arrows) indicates CD45R^+^ cellular infiltration. **(B)** Counts of B (CD45R^+^) cells per field of lung tissue of C57BIV6 mice 7 days post-METH and OVA/LPS administration were determined using light microscopy. Bars indicate the average number of CD45R^+^ cells (*n* = 10 images; 10 fields per image; 2 images per mouse; 5 mice per condition) for untreated, METH, OVA, METH + OVA, LPS, and METH + LPS and error bars indicate STDEV Asterisks (*) indicate *P*-value significance (*P < 0.05, **P < 0.01, ***P < 0.001, and ****P < 0.0001) calculated using ANOVA and adjusted by use of the Tukey’s *post-hoc* analysis.

**FIGURE 4 |:**
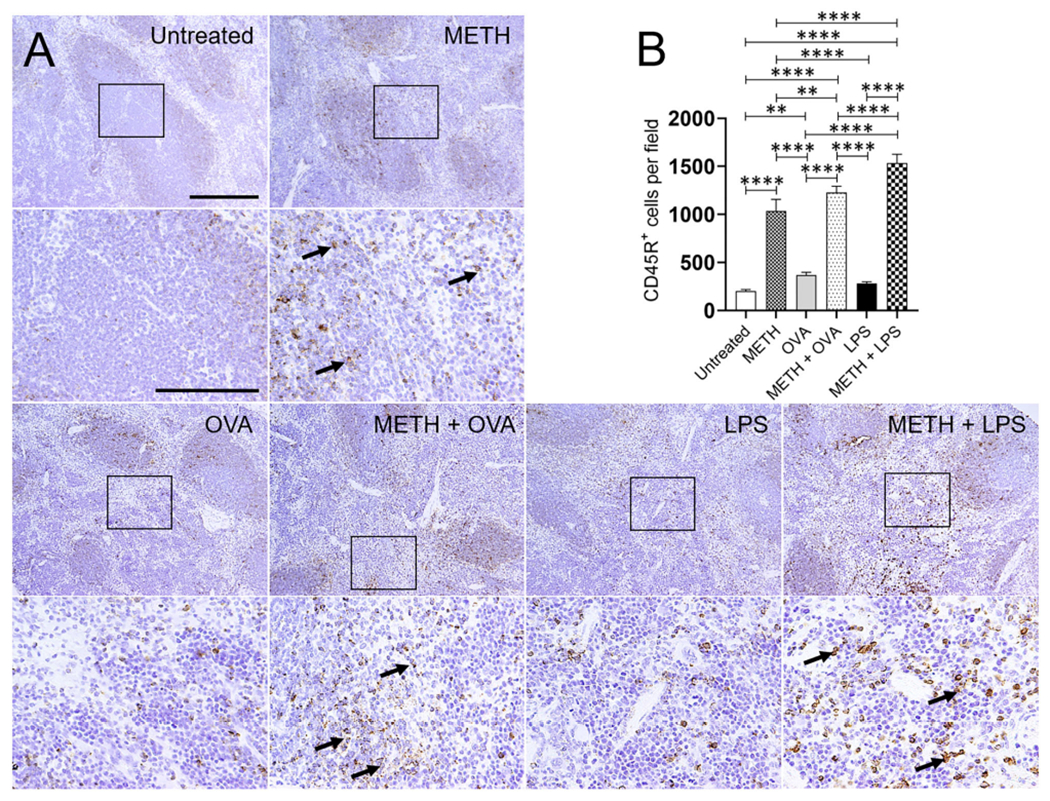
METH modifies CD45R^+^ cells recruitment into the spleen of C57BL/6 mice after OVA or LPS challenge. **(A)** Representative CD45R stained tissue sections of spleens excised from untreated, METH, OVA, METH + OVA, LPS, and METH + LPS-treated animals after 7 days are shown (scale bar, 200 μm; 10 and 40x magnification). Black rectangular box delineates the area magnified. Brown staining (black arrows) indicates CD45R^+^ cellular infiltration. **(B)** Counts of CD45R^+^ cells per field of splenic tissue of C57BL/6 mice 7 days post-METH and OVA/LPS administration were determined using light microscopy. Bars indicate the average number of CD45R^+^ cells (*n* = 10 images; 10 fields per image; 2 images per mouse; 5 mice per condition) for untreated, METH, OVA, METH + OVA, LPS, and METH + LPS and error bars indicate STDEV Asterisks (*) indicate *P*-value significance (**P < 0.01, ****P < 0.0001) calculated using ANOVA and adjusted by use of the Tukey’s *post-hoc* analysis.

Immunohistochemistry analyses of lung and splenic tissue were performed to validate the results obtained by flow cytometry. Histological images of the lungs (Figure 3) and spleen (Figure 4) also demonstrated considerable CD45R^+^ cell recruitment differences between METH, METH + OVA, and METH + LPS-treated mice and animals of the untreated, OVA, and LPS groups. The images of pulmonary and splenic tissue removed from METH, METH + OVA, and METH + LPS-treated animals evinced substantial infiltration (black arrows; brown staining) of CD45R^+^ cells (Figures 3A, 4A), therefore, confirming the flow cytometry analyses. Then, we counted the numbers of B cells infiltrating into the lungs (Figure 3B) and spleen (Figure 4B) of C57BL/6 mice 7 days post-Ag challenge. In the lungs, mice treated with METH had the highest B cell recruitment (Figure 3B). METH (*P* < 0.0001), LPS (*P* < 0.01), or METH + LPS (*P* < 0.0001)-treated rodents had significantly higher numbers of CD45R^+^ cells than untreated animals. Animals treated with METH + OVA (*P* < 0.001), LPS (*P* < 0.0001), and METH + LPS (*P* < 0.0001) exhibited higher B cell infiltration relative to the OVA-treated group. Also, METH + LPS-treated animals showcased higher B lymphocyte infiltration compared to the METH + OVA (*P* < 0.01) and LPS (*P* < 0.05) groups. In spleen, METH + LPS-treated mice demonstrated the highest CD45R^+^ cell recruitment numbers (Figure 4B). Mice treated with METH alone or METH and Ag sensitized showed higher B cell infiltration compared to untreated or Ag challenged groups (*P* < 0.0001). Finally, OVA-treated rodents displayed higher lymphocyte infiltration than untreated mice (*P* < 0.01). Taken together, we concluded that METH administration prolongs the infiltration of B cells into the lungs and spleen of C57BL/6 mice 7 days post-sensitization.

### METH Influences Ab Production in C57BL/6 Mice Sensitized With OVA and LPS

**FIGURE 5 |:**
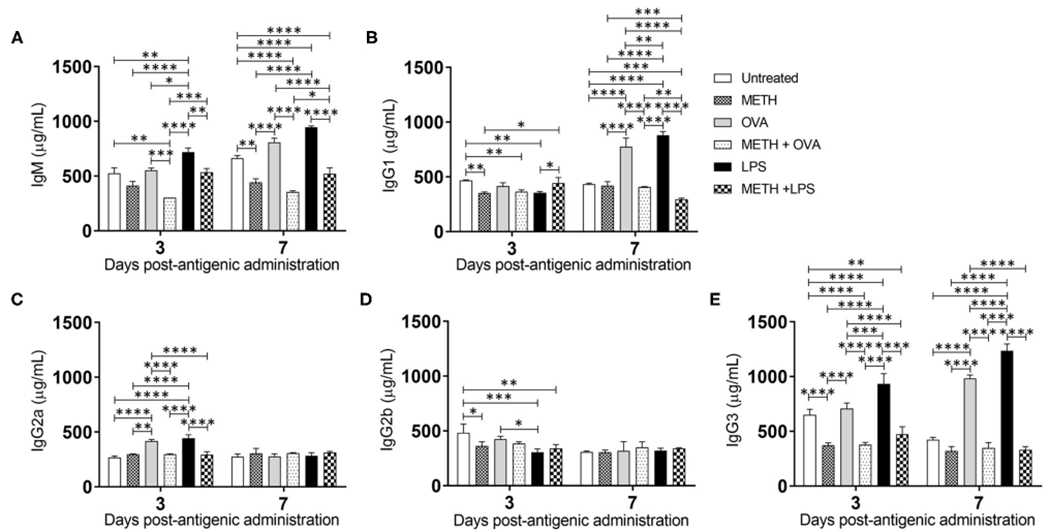
METH alters antibody production in mice challenged with OVA and LPS. **(A)** Immunoglobulin (Ig) M, **(B)** IgG_1_, **(C)** IgG_2a_, **(D)** IgG_2b_, and **(E)** IgG_3_ levels were measured by ELISA in serum of untreated or METH-treated mice (*n* = 5 mice per condition per day) sensitized with OVA or LPS 3- and 7-days post-Ag exposure. Bars represent the average Ab levels for each condition and error bars indicate STDEV Asterisks (*) indicate *P*-value significance (*P < 0.05, **P < 0.01, ***P < 0.001, and ****P < 0.0001) calculated using ANOVA and adjusted by use of the Tukey’s *post-hoc* analysis. The experiments shown were performed twice, and similar results were obtained each time.

Since METH distributes through most organs after its consumption (Volkow et al., 2010), we assessed the levels of Ab isotypes IgM, IgG_1_, IgG_2a_, IgG_2b_, and IgG_3_ in mouse serum after METH treatment and Ag challenge (Figure 5). IgM is expressed as a monomer on the surface of B cells and secreted as a pentamer upon B cell activation with high avidity. IgM is responsible for eliminating Ag in the early stages of humoral immunity before there is sufficient long-lasting IgG. LPS-treated mice showed the highest IgM production on days 3 (716.6 μg/mL) and 7 (946.8 μg/mL) post-Ag challenge (Figure 5A). On day 3, METH + OVA-treated rodents (303.5 μg/mL) had lower IgM levels than untreated (524.6 μg/mL; *P* < 0.01), OVA (552.8 μg/mL; *P* 0.001), LPS (716.6 μg/mL; *P* < 0.0001), and METH + LPS (535.3 μg/mL; *P* < 0.001) groups. On day 7, mice treated with METH (442.4 μg/mL; *P* < 0.01 and *P* < 0.0001), METH + OVA (355.7 μg/mL; *P* < 0.0001), and METH + LPS (520.4 μg/mL; *P* < 0.0001) exhibited lower IgM production compared to all the non-METH treated groups (untreated: 662.4 μg/mL; OVA: μg/mL; LPS: 946.8 μg/mL). Similarly, we measured the levels of IgG isotype classes which provide the most of Ab-based immunity against invading pathogens (Figures 5B–E). On day 3, METH (352.1 μg/mL; *P* < 0.01), METH + OVA (365.9 μg/mL; *P* 0.01), and LPS (356.2 μg/mL; *P* < 0.05)-treated mice evinced lower IgG_1_ levels than untreated mice (Figure 5B). In contrast, METH + LPS (443.6 μg/mL)-treated animals demonstrated higher IgG_1_ production compared to METH (352.1 μg/mL; *P* < 0.05) and LPS (356.2 μg/mL; *P* < 0.05)-treated groups. On day 7, OVA (774.6 μg/mL) and LPS (881.1 μg/mL)-treated animals duplicated the amount of IgG_1_ in serum compared to the levels produced on day 3 (OVA: 414.1 μg/mL and LPS: μg/mL). Animals in the OVA and LPS groups produced the highest levels of IgG_1_ (*P* < 0.0001). METH impaired IgG_1_ production in all the rodent Ag sensitized, with METH + LPS (294.1 μg/mL; *P* < 0.001; 0.0001) having the lowest levels in serum. Statistical differences in IgG_2a_ (Figure 5C) and IgG_2b_ (Figure 5D) levels were only recorded between the groups of animals 3 days post-Ag challenge. OVA (418.4 μg/mL) and LPS (442 μg/mL)-treated mice exhibited significantly higher levels of IgG_2a_ compared to all the other experimental groups (untreated: 267.5 μg/mL, *P* < 0.0001; METH: 296.4 μg/mL, *P* < 0.01 and 0.0001; METH + OVA: 298.4 μg/mL, *P* < 0.0001; METH + LPS: 292.3 μg/mL, *P* < 0.0001) (Figure 5C). Nevertheless, untreated mice (481.7 μg/mL) had significantly higher IgG_2b_ levels than animals treated with METH (363 μg/mL; *P* < 0.05), LPS (305.8 μg/mL; *P* < 0.001), and METH + LPS (340.4 μg/mL; *P* < 0.01) (Figure 5D). LPS-treated mice displayed lower IgG_2b_ in the serum relative to OVA-treated rodents (425.7 μg/mL; *P* < 0.05). Furthermore, all the rodent groups treated with METH (METH: 373.6 μg/mL; METH + OVA: 377.4 μg/mL; METH + LPS: 472.8 μg/mL) demonstrated a significant reduction in serum IgG_3_ levels relative to the groups that were not injected with the drug (untreated: 650.9 μg/mL, *P* < 0.01 and *P* < 0.0001; OVA: 707.4 μg/mL, *P* < 0.0001; LPS: 933 μg/mL, *P* < 0.0001) 3 days post-Ag challenge (Figure 5E). Animals treated with METH + OVA (348.4 μg/mL) or METH + LPS (330.7 μg/mL) showed considerably lower production of IgG_3_ when compared to those mice only challenged with the Ags (OVA: 984 μg/mL, *P* < 0.0001; LPS: 1,235.2 μg/mL, *P* < 0.0001) (Figure 5E). These findings suggest that METH suppresses adaptive humoral immunity by compromising B cell activation and immunoglobulin production.

### METH Impairs the Distribution of IgM on the Surface of Human BJAB Cells

**FIGURE 6 |:**
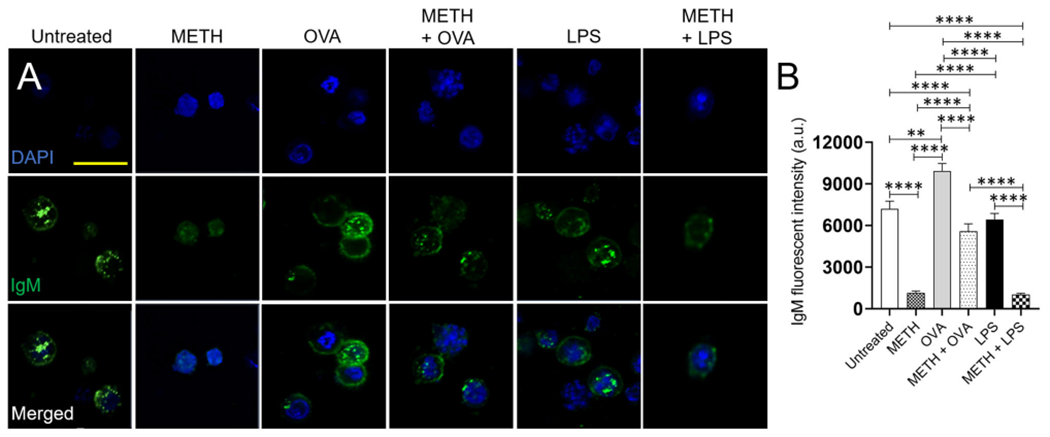
METH reduces the IgM distribution and expression on the surface of human BJAB cells after Ag challenge. **(A)** Immunofluorescent images show the distribution of IgM (green; anti-IgM conjugated to Alexa Fluor 488) on the surface of untreated and treated BJAB cells with METH, OVA, METH + OVA, LPS, and METH + LPS. Nuclei of B cells were stained in blue with dapi. Scale bar, 20 μm. **(B)** The IgM fluorescent intensity of untreated and treated BJAB cells with METH, OVA, METH + OVA, LPS, and METH + LPS was analyzed. Bars represent the mean of 10 cell measurements and error bars indicate STDEV. Asterisks (*) indicate *P* value significance (**P < 0.01, ****P < 0.0001) calculated using ANOVA and adjusted by use of the Tukey’s *post-hoc* analysis. The experiments shown were performed twice, and similar results were obtained each time.

BJAB cells synthesize both cell-surface IgM and secretory IgM (Singer and Williamson, 1980). Since METH altered B cell-mediated immunity *in vivo*, we determined the impact of the drug on the distribution and expression of IgM on the surface of BJAB cells using fluorescent microscopy (anti-IgM-conjugated to Alexa Fluor 488, green; dapi, blue) (Figure 6A). The quantification of fluorescent IgM intensity demonstrated that BJAB cells treated with METH, METH + OVA, or METH + LPS had reduced immunoglobulin on their surfaces compared to untreated or OVA and LPS-treated cells (*P* < 0.0001) (Figures 6A,B). B-like cells incubated with OVA displayed the higher distribution of IgM on their surface than untreated (*P* < 0.01) and LPS-exposed (*P* < 0.0001) cells. Similarly, BJAB cells incubated with METH + OVA exhibited higher IgM distribution than METH-treated cells (*P* < 0.0001). To exclude the possibility that the differences in IgM distribution on the surface of B-like cells was not because of the Ab secretion into the medium, we attempted to determine the concentration of secreted IgM having inconclusive results. Thus, as proof of principle, we performed western blot analysis and only tested BJAB cells treated with METH or METH + OVA due to the differences in IgM distribution observed in the cells of these groups in the immunofluorescence experiments. Our results indicate that BJAB cells treated with METH + OVA had significantly reduced IgM expression (Supplementary Figures1A,B) (*P* < 0.05). Our data indicate that METH inhibits the distribution and expression of IgM on the surface of B cells and these effects might have formidable consequences in the establishment of the adaptive humoral immune response.

## DISCUSSION

Our findings demonstrated that METH prolongs B cell infiltration into the lungs and spleen of mice after 7 days of sensitization with each Ag. Since B cells extended recruitment to pulmonary and splenic tissue is independent of OVA or LPS exposure, it is conceivable that the accumulation of these lymphocytes is due to the recognition of METH as an Ag. This postulate is supported by studies describing the generation of METH-specific monoclonal Abs, which have been proposed as potential therapeutics for the treatment of METH addiction (Owens et al., 2011; Peterson et al., 2014; Hambuchen et al., 2016). Likewise, chronic drug use has been associated with B cell accumulation, an increase in the incidence of autoAbs, and the development of autoimmunity (Simonovska et al., 2016). For example, long-term METH use causes chronic kidney disease due to the deposition of IgM and C3 complement (Jones and Rayner, 2015). It is possible that the maintenance of B cells in the lungs and spleen is related to the accumulation of METH in these tissues and the sensitivity of other cells to the drug. METH induces apoptosis in macrophages (Aslanyan et al., 2019) and T cells (Potula et al., 2010). However, B cell viability *in vitro* is only affected by a significantly high METH concentration (100 μM) or four times the concentration (25 μM) used in our studies (House et al., 1994). Because METH interferes with phagocytic and cell-mediated responses, this could result in the host being left without immunological responses from neither innate nor acquired immunity. Even though we and others recently showed that METH compromises the toll-like receptor (TLR4)/MD2 complex signaling pathways, alters NF-κB activation, and interferes with the synthesis of pro-inflammatory cytokines by microglia after LPS exposure (Wang et al., 2019; Vargas et al. (2020)), there is no data available on the impact of this psychostimulant in the regulation of TLR4 signaling by B cells and its role in the modulation of Ab production or other immune effector molecules. TLRs are expressed on B cells, and TLR signaling in these lymphocytes contributes to Ab-mediated immunity and autoimmunity (Mocsai et al., 2010). The SYK tyrosine kinase is necessary for signaling from the B cell Ag receptor (BCR), and so for Ab responses to T-dependent and -independent Ags (Ackermann et al., 2015). B cell exposure to LPS stimulates TLR4 signaling via the BCR, leading to activation of SYK, ERK, and AKT and also through MYD88 resulting in the activation of NF-κB (Schweighoffer et al., 2017). It is possible that METH causes similar TLR4 signaling defects in B cells, skewing the production of inflammatory mediators resulting in faulty Ab production and significant tissue damage via autoimmunity, massive and sustained immune cell infiltration, or flawed responses to microbial infection. Hence, potential investigations elucidating the relationship of METH use, B cell activation through TLRs after exposure to Ags or infection, and Ab-mediated immunity or autoimmunity are warranted.

METH has important implications on Ab production (Wey et al., 2008; Martinez et al., 2009), phagocytic responses (Mihu et al., 2015), and the fate of pathogens (Aslanyan et al., 2019) during infection. In addition, Ab isotype might be critical in stimulating the phagocytic cell migration and antimicrobial effector functions. Using the AIDS-related encapsulated fungus *Cryptococcus neoformans* as a model microorganism of microbe-phagocytic cell interactions, we previously demonstrated that IgM promotes complement-mediated phagocytosis of the fungus by macrophages (Aslanyan et al., 2017). Fungal cells grown with complement and IgM in the presence of METH showed higher number of cells per aggregate, a plausible description for their greater engulfment by phagocytes. The pentameric structure of IgM has ten Fab binding sites per molecule, being the most effective immunoglobulin for microbial aggregation and elimination by phagocytic cells. IgM facilitated fungal cell killing by macrophages, increased the phagosome’s pH, and promoted the production of nitric oxide intracellularly. T-cell independent Ags can stimulate the early production of IgM Abs. Our results indicate that IgM is significantly produced 3 days post LPS challenge and the levels are similarly high in untreated and METH + LPS-treated animals. Thus, it is plausible that IgM stimulate the antimicrobial functions of METH-resistant macrophages against opportunistic microorganisms.

IgG is the most abundant Ab in blood and extracellular fluid, enabling it to combat infection of body tissues. We observed high IgG_2a_ and IgG_3_ levels in the serum of mice treated with OVA and LPS after 3 days, although rodents treated with the drug had reduced levels of these subclasses. METH-treated animals challenged with LPS exhibited early higher IgG_1_ levels in serum than LPS-treated animals. IgG_1_ and IgG_3_ were the only IgG subclasses abundantly produced by mice sensitized with OVA and LPS after 7 days and significantly reduced by METH injection. IgG_1_ and IgG_3_ activate complement and bind to Fc receptors on phagocytic cells with high affinity. We recently investigated the efficacy of a specific IgG_1_ to *C. neoformans* in phagocytosis and Ag processing by macrophages (Aslanyan et al., 2019). In contrast to the results obtained with IgM, we found that METH prevents IgG_1_-mediated phagocytosis of cryptococcal cell by macrophages, mostly by reducing the expression of Fcγ receptors on phagocyte membranes. METH interfered with phagocytic cells’ phagosomal maturation and nitric oxide production, resulting in impaired fungal control. The opposite phagocytic cell responses obtained against *C. neoformans* in presence of IgM or IgG can be explained by users who develop an IgM response to cocaine after recurrent recreational consumption to this drug are considerably less likely to synthesize high concentrations of IgG Abs (Orson et al., 2013). The impairment is probably linked to recreational cocaine exposure initiation of a T cell-independent immune response (Orson et al., 2013). Additionally, METH might induce the production of non-protective and disease enhancing Abs (Martinez et al., 2009). Mice injected with METH and infected with the fungal pathogen *Histoplasma capsulatum* have demonstrated a skew high IgG_2b_ levels resulting in lethal histoplasmosis (Martinez et al., 2009). In humans, high IgG_3_ production in intravenous drug users has been associated with chronic B cell activation, excessive systemic inflammation, and increased HIV exposure (Piepenbrink et al., 2016).

METH-treated rodents sensitized with OVA and LPS had lower levels of IgM and total IgG. Our results are similar to those obtained by Wei et al., who showed that METH significantly diminished the synthesis of OVA-specific IgM, IgG_1_, and IgG_2a_ (Wey et al., 2008). These results were confirmed *in vitro* via fluorescent microscopy by testing that BJAB cells treated with METH and exposed to OVA or LPS had lower distribution of IgM on their surface than those cells incubated only with the Ag. Although there was a possibility that the decreased IgM receptors on the surface of METH-treated and Ag challenged BJAB was related to the secretion of the Ab molecules to the medium, this possibility was ruled out by a western blot analysis (Supplementary Figure 1) demonstrating a reduced expression of IgM by these B-like cells after exposure to the drug and challenge with OVA. Moreover, total Ab production by B cells might reflect normal biological variation in group differences in association with the acute effects of LPS or indirect bystander effect following OVA on B cell Ig expression/secretion.

In conclusion, we demonstrated that METH compromises humoral immunity in C57BL/6 mice upon Ag challenge. METH accumulation promotes B cell infiltration into tissues and decreases Ab systemic levels. These observations may alter the ability of METH users to generate optimal primary or secondary immune responses to microbial infection, or are at high risk for developing autoimmunity, and should be carefully considered when developing METH addiction management interventions for this growing population. Future studies should emphasize on expanding our knowledge about METH use and the defects this compulsive behavior causes B cell memory and the relationship between autoreactive Abs and organ injury.

## Supplementary Material

Supplementary Figure 1 METH reduces the IgM expression on the surface of human BJAB cells after Ag challenge. **(A)** The expression of IgM in BJAB cells was determined by western blot analysis. B cells were cultured with 25 μM METH for 2 h, followed by an incubation in the absence (METH) or presence of 10 μg/mL OVA (M+O) for 24 h. GAPDH was used as a housekeeping gene control. **(B)** The levels of expression of IgM were measured by determining the relative intensity ratios. Individual band intensities from the western blot in **(A)** were quantified using ImageJ software. The GAPDH protein was used as a reference to determine the relative intensity ratios shown in **(B)**. Bars represent the mean of three independent experiments (*n* = 3) and error bars indicate standard deviations. σ indicates *P*-value significance (*P* < 0.05) calculated using student’s *t*-test analysis.

## Data Availability

The raw data supporting the conclusions of this article will be made available by the authors, without undue reservation.
