# Supplementary figures and images for: Methamphetamine Compromises the Adaptive B Cell-Mediated Immunity to Antigenic Challenge in C57BL/6 Mice
Source: Front Toxicol. 2021 Mar 15;3:629451. doi: 10.3389/ftox.2021.629451 (PMC8186300; doi:10.3389/ftox.2021.629451)

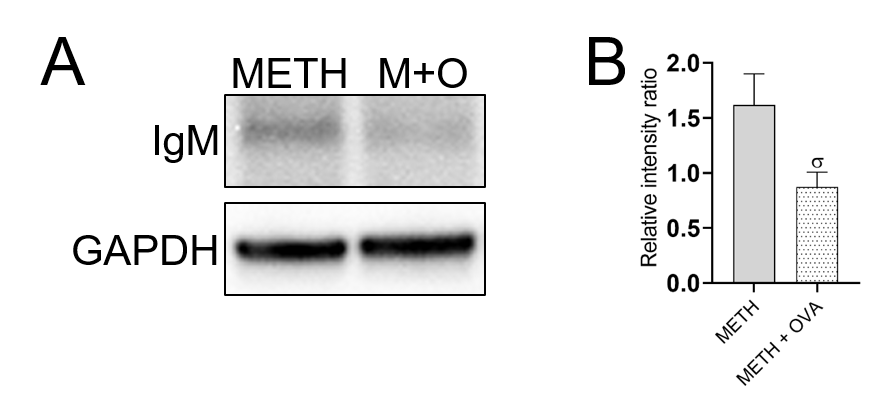

Supplement: Supplementary Figure 1 — METH reduces the IgM expression on the surface of human BJAB cells after Ag challenge. (A) The expression of IgM in BJAB cells was determined by western blot analysis. B cells were cultured with 25 μM METH for 2 h, followed by an incubation in the absence (METH) or presence of 10 μg/mL OVA (M+O) for 24 h. GAPDH was used as a housekeeping gene control. (B) The levels of expression of IgM were measured by determining the relative intensity ratios. Individual band intensities from the western blot in (A) were quantified using ImageJ software. The GAPDH protein was used as a reference to determine the relative intensity ratios shown in (B). Bars represent the mean of three independent experiments (n = 3) and error bars indicate standard deviations. σ indicates P-value significance (P < 0.05) calculated using student's t-test analysis. [file Image_1.TIF]
